# Der okkulte Fremdkörper im Larynx

**DOI:** 10.1007/s00106-021-01043-y

**Published:** 2021-04-08

**Authors:** David Hortobagyi, Wolfgang Anderhuber, Hannaleena Tervonen

**Affiliations:** Hals-Nasen-Ohren-Universitätsklinik, Medizinische Universität Graz, Auenbruggerplatz 26, 8036 Graz, Österreich

## Anamnese

Eine 61-jährige Frau wurde mit Zuweisung vom Zahnarzt wegen eines Fremdkörpergefühls in unserer HNO-Notaufnahme vorstellig. Sie berichtete, im Rahmen der zahnärztlichen Behandlung sei es zu einem Verlust einer Schraube ihrer Zahnprothese gekommen, seither hätte sie das Gefühl, die Schraube würde in ihrem Hals feststecken. Ansonsten war die Patientin völlig beschwerdefrei, insbesondere konnten keinerlei respiratorische Symptome wie Dyspnoe erhoben werden.

## Befunde und Diagnose

**Figure 1:**
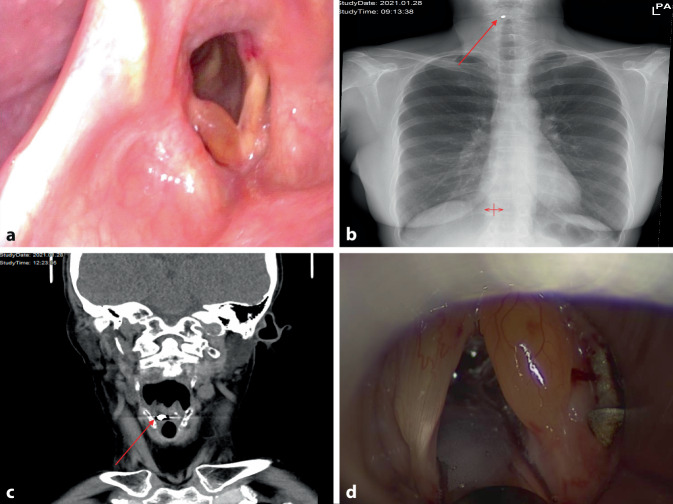

Bei der HNO-ärztlichen Untersuchung mit Inspektion der Hypopharynx- und Larynxebene mithilfe eines flexiblen Endoskops fiel eine ödematöse Schwellung der rechten Stimmlippe auf (Abb. 1a), die laut Patientin jedoch schon bekannt und unter regelmäßiger Observanz war. Der übrige Larynxbefund, insbesondere die Beweglichkeit beider Stimmlippen, zeigte sich unauffällig. Da kein Fremdkörper ersichtlich war, erfolgte eine endoskopische Tracheoskopie. Nachdem die Trachea bis zur Bifurkation frei war, wurde das Endoskop durch den rechten Sinus piriformis in den Ösophagus vorgeschoben. Ein Fremdkörper wurde auch hierbei nicht gesichtet.

Da die Patientin weiterhin über anhaltende Beschwerden klagte, wurde ein Videoschluckaktröntgen (VISA) in Erwägung gezogen, aber aufgrund einer fraglichen Kontrastmittelallergie wurde von dieser Untersuchung Abstand genommen. In den stattdessen angeordneten Röntgenaufnahmen des Halses, Thorax und Abdomens zeigte sich ein röntgendichter Fremdkörper von 1,2 cm × 0,6 cm auf Ebene des Kehlkopfs beziehungsweise des Hypopharynx (Abb. 1b).

Nun entschloss man sich zu einer Panendoskopie inklusive Mikrolaryngoskopie mit einem starren Rohr mit der Annahme, dass die Schraube am Eingang der Speiseröhre eingeklemmt war. Auch hier blieb der Fremdkörper weiterhin unentdeckt. Es wurde vermutet, dass der Fremdkörper sich durch die anästhesiologische Relaxierung gelöst hatte und in den Magen gelangt seien könnte.

Am Folgetag berichtete die Patientin über ein weitgehend unverändertes Fremdkörpergefühl auf Höhe des Kehlkopfs. Erst die Computertomographie (CT) vom Hals bis zum Abdomen zeigte eindeutig eine Schraube im rechten Sinus Morgagni (Abb. 1c).

## Wie lautet Ihre Diagnose?

**Diagnose:** Fremdkörper im Sinus Morgagni

## Therapie und Verlauf

Eine Kontrollendoskopie mit einem flexiblen Endoskop war neuerlich ergebnislos, deshalb entschied man sich für eine Mikrolaryngoskopie in Vollnarkose. Durch das Verdrängen der rechten Taschenfalte mit dem starren Rohr, konnte die Schraube schließlich gefunden und geborgen werden (Abb. 1d).

Die Aspiration von Fremdkörpern ist eine sehr seltene, aber möglicherweise lebensbedrohliche Entität. Bei Erwachsenen betrifft es vor allem Patienten mit neuromuskulären Erkrankungen, verändertem Geisteszustand oder Vergiftungen. Berichten zufolge können auch zahnärztliche Eingriffe mit örtlicher Betäubung zu einer versehentlichen Fremdkörperinhalation führen. Die Symptome hängen von der Lage und Größe ab und reichen von Stridor und Atemnot mit akutem Atemversagen bis hin zu alleinigem Husten oder, sind in seltenen Fällen völlig symptomlos [2, 5].

Der Umfang vorhandener Literatur mit hohem Evidenzlevel zum Vorgehen bei Patienten mit Fremdkörperaspiration ist äußerst spärlich. Dies liegt in der Natur von Notsituationen, in denen eine Durchführung randomisiert kontrollierter Studien kaum möglich ist. Es existieren Empfehlungen, diese beruhen jedoch hauptsächlich auf Fallberichten und Fallserien.

Eine detaillierte Anamnese und eine Übersicht über die klinischen Symptome sind die ersten Schritte zur Diagnose. Kadmonet et al. entwickelten ein Bewertungssystem für die Diagnose der Fremdkörperaspiration, das auf Parametern wie Alter, Anzeichen von Atemnot und Röntgenbefund basiert. Letzteres ist, obwohl weniger sensitiv als eine CT, aufgrund der geringen Strahlenbelastung häufig die erste durchgeführte Bildgebungsmodalität.

Obwohl ein VISA in den Empfehlungen nicht erwähnt wird, könnte es bei Patienten, bei denen der Verdacht besteht, die Symptome könnten auf einen Fremdkörper zurückzuführen sein, sinnvoll sein. Dies mag auf den ersten Anschein überraschend klingen, denn die Beurteilung der Atemwege wird dadurch nicht verbessert. Allerdings sollte man nicht außer Acht lassen, dass man sich im klinischen Alltag häufig nicht sicher ist, ob ein etwaiger Fremdkörper in den Atemwegen oder im Bereich der Speisewege steckt. Sollte ein Fremdkörper im Bereich der Speisewege liegen, so kann dieser durch eine Kontrastmittelaussparung im VISA auch ersichtlich werden, selbst wenn er nicht röntgendicht ist. Und zuletzt vermuten wir, dass ein VISA eine klarere Abgrenzbarkeit zwischen Larynx und Hypopharynx ermöglicht hätte. So wäre die Lokalisation des Fremdkörpers mit einer geringeren Strahlenbelastung als eine CT erleichtert gewesen. Daher sollte beim Verdacht auf Fremdkörperaspiration/-ingestion ein VISA erwogen werden, sofern keine Kontraindikationen wie beispielsweise eine Perforation vorliegen [1, 2, 4, 5].

Therapeutisch steht der Schutz der Atemwege im Vordergrund. Bei instabilen Patienten, die bei Bewusstsein sind, sollte als Erstmaßnahme das Heimlich-Manöver durchgeführt werden.

In Fällen, in denen sich der Patient in einem stabilen Zustand befindet und der Fremdkörper entdeckt werden kann, sollte seine Entfernung so schnell wie möglich, zumindest innerhalb von 6 h, erfolgen. Hierbei spielt insbesondere die flexible Endoskopie eine besondere Rolle, da sie neben ihrer diagnostischen Bedeutung bei Vorhandensein eines Arbeitskanals auch die Extraktion des Fremdkörpers ermöglicht. Wegen ihrer relativ leichten Handhabung und der Möglichkeit des Verzichts auf eine Narkose hat die flexible Endoskopie in vielen Fällen die starre abgelöst. Die Verwendung eines starren Endoskops ist jedoch in einigen Situationen immer noch gerechtfertigt. Beispielsweise wenn die Schleimhaut vor scharfen Gegenständen geschützt werden muss oder um die Speiseröhre besser zu entfalten und so eine gründlichere Übersicht zu erhalten. Aus letzterem Grund wurde sie auch im vorliegenden Fall indiziert [2, 3, 5].

## Fazit für die Praxis

- Bei einer Fremdkörperaspiration steht die Sicherung der Atemwege im Vordergrund der Therapie.
- Jeder Fremdkörper sollte so schnell wie möglich entfernt werden.
- Beim Verdacht auf eine Fremdkörperaspiration/-ingestion sollte, sofern keine Kontraindikation (z. B. Perforation) vorliegt, ein VISA erwogen werden.
